# Short-Term Cardiovascular Response to Short-Radius Centrifugation With and Without Ergometer Exercise

**DOI:** 10.3389/fphys.2018.01492

**Published:** 2018-11-13

**Authors:** Ana Diaz-Artiles, Thomas Heldt, Laurence R. Young

**Affiliations:** ^1^Department of Aerospace Engineering, Texas A&M University, College Station, TX, United States; ^2^Institute for Medical Engineering and Science and Department of Electrical Engineering and Computer Science, Massachusetts Institute of Technology, Cambridge, MA, United States; ^3^Department of Aeronautics and Astronautics, Massachusetts Institute of Technology, Cambridge, MA, United States

**Keywords:** artificial gravity, orthostatic intolerance, human experiments, spaceflight deconditioning, spaceflight countermeasure

## Abstract

Artificial gravity (AG) has often been proposed as an integrated multi-system countermeasure to physiological deconditioning associated with extended exposure to reduced gravity levels, particularly if combined with exercise. Twelve subjects underwent short-radius centrifugation along with bicycle ergometry to quantify the short-term cardiovascular response to AG and exercise across three AG levels (0 G or no rotation, 1 G, and 1.4 G; referenced to the subject’s feet and measured in the centripetal direction) and three exercise intensities (25, 50, and 100 W). Continuous cardiovascular measurements were collected during the centrifugation sessions using a non-invasive monitoring system. The cardiovascular responses were more prominent at higher levels of AG and exercise intensity. In particular, cardiac output, stroke volume, pulse pressure, and heart rate significantly increased with both AG level (in most of exercise group combinations, showing averaged increments across exercise conditions of 1.4 L/min/g, 7.6 mL/g, 5.22 mmHg/g, and 2.0 bpm/g, respectively), and workload intensity (averaged increments across AG conditions of 0.09 L/min/W, 0.17 mL/W, 0.22 mmHg/W, and 0.74 bpm/W respectively). These results suggest that the addition of AG to exercise can provide a greater cardiovascular benefit than exercise alone. Hierarchical regression models were fitted to the experimental data to determine dose-response curves of all cardiovascular variables as a function of AG-level and exercise intensity during short-radius centrifugation. These results can inform future studies, decisions, and trade-offs toward potential implementation of AG as a space countermeasure.

## Introduction

Astronauts undergo important physiological deconditioning in space due to the weightless environment. Bone loss, muscle atrophy, cardiovascular deconditioning, or neurovestibular alterations are some of the most common issues experienced during space missions (Clément, 2005; Buckey, 2006). The observed changes in cardiovascular performance have been attributed to the loss of hydrostatic pressure gradients in microgravity (Charles and Lathers, 1991; Williams et al., 2009), causing a series of physiological adaptations, including a fluid shift from the lower extremities to the upper part of the body, a decrease in circulating blood volume, cardiac atrophy, an increase in venous compliance, a reduction of the baroreflex sensitivity (Clément and Bukley, 2007), and other alterations in autonomic function (Mandsager et al., 2015). Oxidative stress (Dhalla et al., 2000), radiation (Baker et al., 2011), and elevated CO_2_ levels (Sliwka et al., 1998) may also affect cardiovascular regulation. Previous in-flight investigations have shown significant changes in hemodynamics during both short- (Norsk et al., 2006; Verheyden et al., 2009; Eckberg et al., 2010; Norsk, 2014; Norsk et al., 2015), and long-duration (Baevsky et al., 2007; Verheyden et al., 2009; Hughson et al., 2012; Norsk et al., 2015) spaceflight. This general adaptation of the cardiovascular system to weightlessness, especially if combined with detrimental musculoskeletal changes (LeBlanc et al., 2007; Fitts et al., 2010), may lead to orthostatic intolerance (Buckey, 2006) and a significant reduction in work capacity (Levine et al., 1996) when re-exposure to a significant gravitational environment, like on Earth, occurs.

Currently, there are several countermeasures in place that seek to mitigate the detrimental effects of weightlessness. The introduction of the new Advanced Resistance Exercise Device (ARED) in 2008 has resulted in an attenuation in bone loss when coupled with other countermeasures such as adequate energy intake and vitamin D (Smith et al., 2012), or bisphosphonates (LeBlanc et al., 2013). In general, these countermeasures are specific to individual physiological systems. Cardiovascular countermeasures, for example, have included aerobic and resistive exercise (Buckey, 2006; Trigg, 2013), Lower Body Negative Pressure (LBNP) (Charles and Lathers, 1994; Clément and Bukley, 2007), fluid loading (Clément and Bukley, 2007), intra-vehicular activity suits (Kozlovskaya et al., 1995; Kozlovskaya and Grigoriev, 2004; Waldie and Newman, 2011), landing compression garments (Platts et al., 2009), and nutrition and dietary supplements (Buckey, 2006; Smith et al., 2012). To date, however, countermeasures have failed to consistently preserve pre-flight levels of physiological function, despite the significant crew time that needs to be allocated to them (Clément and Pavy-Le Traon, 2004; Kozlovskaya and Grigoriev, 2004; Buckey, 2006; Clément and Bukley, 2007; Trappe et al., 2009; Lee et al., 2015). Consequently, about two-thirds of returning Shuttle astronauts experienced some degree of orthostatic intolerance (Buckey et al., 1996; Lee et al., 2015), with one out of every four astronauts failing to complete a 10-min stand test on landing day due to light-headedness, palpitations, and syncope (Williams et al., 2009). The incidence of orthostatic intolerance is even greater after longer missions in the ISS, where the proportion of astronauts that could not complete a 10-min orthostatic tilt test is about 33% (Lee et al., 2015). New approaches, possibly combining novel and current countermeasures, are needed for longer missions in the future, such as a trip to the surface of Mars, where astronauts will not have the ground support and resources usually provided on Earth after landing.

Artificial gravity (AG) has been proposed as a promising multisystem countermeasure (Paloski and Charles, 2014; Clément G.R. et al., 2016; Clément, 2017). By intermittently creating a gravitational gradient along the major body axis, AG has the potential to prevent multiple aspects of human deconditioning from occurring during long exposure to weightlessness. Different physiological systems, including the musculoskeletal, cardiovascular, neurovestibular, and immune systems could benefit at the same time from AG exposure while in space (Buckey, 2006; Clément and Bukley, 2007; Paloski and Charles, 2014; Clement et al., 2015; Linnarsson et al., 2015). Furthermore, the benefits of centrifugation could be enhanced if combined with exercise. In addition to its direct contribution to muscle and cardiovascular conditioning, exercise during AG increases tolerance to centrifugation via the muscle pump, protecting astronauts from fainting (Clément and Bukley, 2007; Kaderka et al., 2010). In particular, lower body cycling exercise using an ergometer device mounted on a short-radius centrifuge while rotating has been shown to be effective in preventing cardiovascular deconditioning (Greenleaf et al., 1997; Iwase et al., 2002; Evans et al., 2004; Katayama et al., 2004; Iwase, 2005; Iwasaki et al., 2005; Stenger et al., 2007; Yang et al., 2007, 2010). AG therefore has the potential to provide a greater overall physiological benefit for a given amount of exercise and crew time (Paloski and Charles, 2014; Clement et al., 2015). Similarly, AG could provide similar physiological benefit with less amount of exercise and crew time. Despite the potential benefits and the increasing interest in this area from the scientific community (Paloski and Young, 1999; Young et al., 2009; Paloski and Charles, 2014), many questions still remain concerning its implementation. Aspects such as the appropriate gravity level, centrifuge configuration, radius, angular velocity, exposure time, exercise modality, exercise protocol, or safety concerns are still unanswered, and require further investigation (Clément G. et al., 2016). Of particular interest is to understand the relationship between gravitational dose and physiological response, which is unknown for most human physiological systems (Clément, 2017).

Recent studies have investigated acute cardiovascular responses *while* being exposed to AG using a short-radius centrifuge. A study conducted at the Institute for Space Medicine and Physiology (MEDES), in Toulouse, France, investigated two gravity levels (1 G and 2 G, measured at the feet), and concluded that centrifugation at 2 G provides similar blood pressure regulatory indices (Verma et al., 2018), and similar cardiovascular and cerebral responses (Goswami et al., 2015a) to standing. Another study conducted at the German Aerospace Institute (DLR) also investigated cardiovascular reactions to centrifugation at 1 and 2 G (measured at the feet) showing apparent gender-specific patterns in their cardiovascular responses (Masatli et al., 2018). In both studies, subjects adopted a supine position with the head located near the center of rotation. However, in all cases subjects were laying down passively, without engaging in any exercise or muscle pump-related contraction.

The objective of our research is to characterize the dynamic cardiovascular response during centrifugation, generated by a short-radius centrifuge, combined with lower-body ergometer exercise in healthy human volunteers. An experimental approach was implemented to investigate the short-term cardiovascular effects of the gravity level and the exercise intensity, and to generate gravitational dose-response curves in a short-radius centrifuge. It was hypothesized that higher levels of AG would increase the stress upon the cardiovascular system and thereby elicit more pronounced cardiovascular reflex responses in order to maintain blood pressure homeostasis. Additionally, it was further hypothesized that these responses would be more apparent when cardiovascular demand increased further at higher exercise intensities.

## Experimental Methods

### Centrifuge Configuration

**FIGURE 1:**
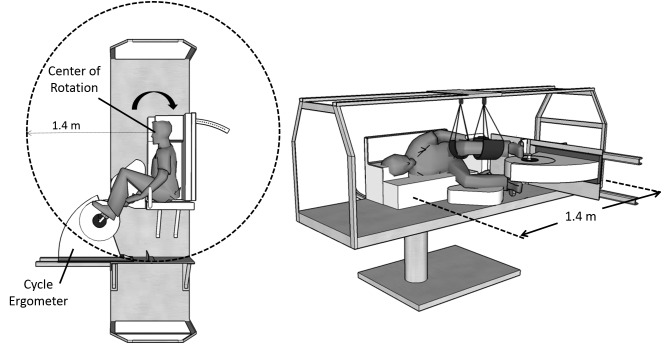
MIT short-radius centrifuge configuration during the experimental sessions. The centrifuge was constrained to a radius of 1.4 m, and a cycle ergometer was used during the centrifugation runs. Subjects were positioned on their right side facing “into the wind.”

The experiments were conducted using the Compact-Radius Centrifuge (CRC) at the Massachusetts Institute of Technology (MIT) (Diaz et al., 2015b). Prior to the study, the centrifuge underwent several modifications, primarily driven by the international flight project “Artificial Gravity with Ergometer Exercise” (AGREE) (Diaz et al., 2015b). This project sought to integrate a short-radius centrifuge with an ergometer exercise device for possible deployment in the Permanent Multipurpose Module (PPM) on-board the International Space Station (ISS). Thus, the new configuration of the MIT centrifuge incorporated the majority of requirements from AGREE, particularly the space limitations and subject configuration, resulting in three main modifications. First, the radius of the centrifuge was constrained to 1.4 m (maximum upper radial limit to fit a centrifuge into the PMM). Second, an ergometer device (Lode BV, Groningen, Netherlands) was incorporated into the centrifuge, allowing subjects to engage in lower-body cycling while being rotated. Finally, subjects were positioned in the right-lateral decubitus position with their head located at the center of the rotation and facing “into the wind,” and their feet strapped to the ergometer device. This positioning not only minimizes motion sickness, but also minimizes lateral knee deflections caused by Coriolis forces while cycling (Duda et al., 2012). If needed, the ergometer position was slightly adjusted to ensure subjects could properly reach the pedals during cycling. The left leg was suspended using adjustable leg straps to facilitate the exercise in the sidewise position. Figure 1 shows a schematic of the MIT centrifuge configuration. Subjects were secured using a three-point seat-belt and monitored continuously using a wireless video camera. A detailed description of the CRC MIT centrifuge configuration and subject positioning is available elsewhere (Diaz et al., 2015a,b).

### Experimental Design and Instrumentation

We implemented a within-subject, counterbalanced, full factorial experimental design, such that all subjects experienced every combination of AG level and exercise intensity. Each subject participated in three experimental sessions, scheduled on three different days within the same week. The experimental sessions were usually scheduled on consecutive days, although due to subjects’ scheduling constraints some sessions occurred every other day. For each subject, the three sessions were scheduled in the morning at approximately the same time to control for possible confounding circadian effects. Additionally, on testing days the subjects were asked to refrain from drinking caffeine and exercising prior to each test session. In each of the three sessions, subjects underwent the same 25-min experimental protocol, under a specific AG level. The AG levels tested were: 0 G (no rotation), 1 G, and 1.4 G. These levels were chosen based on either their relevance to spaceflight (0 and 1 G), or the maximal rotation capacity of the centrifuge (1.4 G). The order of the three sessions (0 G vs. 1 G vs. 1.4 G) was counterbalanced across subjects, meaning that subjects experienced the three AG conditions in different order to counteract for potential carryover effects. The AG levels were measured at the subject’s feet (i.e., the axis of rotation of the ergometer), and they corresponded approximately to rotation rates of 0 rpm (0 G), 28.6 rpm (1 G), and 33.4 rpm (1.4 G). Depending on the ergometer position, these rates were adjusted slightly to ensure that the appropriate gravity level was reached at each subject’s feet.

During the experimental sessions, beat-to-beat cardiac output (CO), stroke volume (SV), pulse pressure (PP), heart rate (HR), mean (MBP), systolic (SBP), and diastolic blood pressure (DBP), and total peripheral resistance (TPR) were continuously determined, recorded, and archived using the Nexfin monitor (Edwards Lifesciences Corporation, Irvine, CA, United States). The only interface with the subject was an inflatable finger cuff that includes an infrared photo-plethysmograph sensor to measure the volume of the finger arteries to calculate the real-time finger pressure waveform. The finger pressure waveform is then transformed to brachial pressure waveform to counteract both the pressure drop to resistance, and the pressure wave amplification in peripheral sites like the fingers (Penaz, 1973; Bogert and van Lieshout, 2005; Eeftinck Schattenkerk et al., 2009; Perel et al., 2011). Blood pressure numerics and HR are directly obtained from the branchial pressure waveform while SV, and thus CO = SV^∗^HR and TPR = MAP/CO, are estimated by the Nexfin CO-trek algorithm and depend on subject’s age, gender, height, and weight (Truijen et al., 2012). Subjects were instructed to keep their hand with the finger cuff at heart level during the entire protocol to avoid pressure changes due to altered hydrostatic effects caused by the rotating environment and the strong gravity gradient present in a short-radius centrifuge. In addition to the cardiovascular recordings, foot-force data using force plates mounted on the pedals (Vernier Software & Technology), and subjective data related to comfort and motion sickness (using an exit survey) were also collected during the experiments, and these results are available elsewhere (Diaz et al., 2015b).

### Artificial Gravity Profile and Exercise Protocol

**FIGURE 2:**
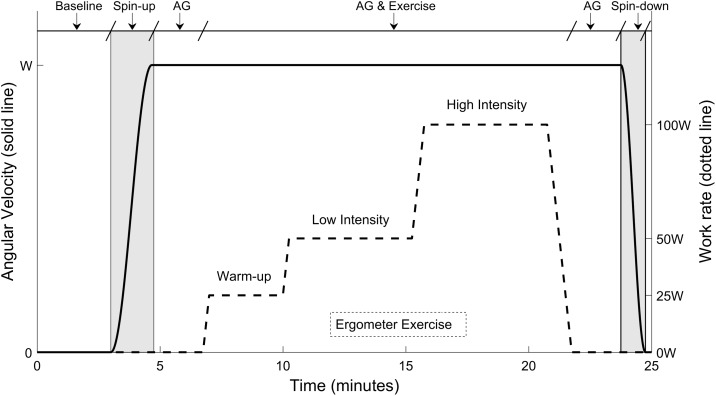
Twenty-five minutes exercise protocol conducted at each experimental session. The protocol included a baseline period (3 min), the spin-up process to the desired G-level (100 s), a first period of AG alone before exercise (125 s), the ergometer exercise period (15-min including work rate transitions), a second period of AG alone after exercise (120 s), and the spin-down process (60 s). The three G-levels tested were 0, 1, and 1.4 G (measured at the feet), corresponding to angular velocities of 0, 28.6, and 33.4 rpm respectively.

While the gravity level varied among experimental sessions, the exercise protocol remained the same and it is shown in Figure 2. Once subjects were positioned on the centrifuge, their cardiovascular variables were recorded during a baseline period of 3 min. Then, the centrifuge either remained motionless (0 G condition) or was accelerated over approximately 100 s to the desired G-level. The acceleration was sufficiently smooth to ensure subjects were comfortable during this process. Approximately 2 min were provided at the end of the acceleration for the transient effects of the spin-up to subside and for subjects to get used to the new gravitational load. Subjects then executed the exercise portion of the testing protocol. Once completed, an additional 2 min were provided for subjects to partially recover from the exercise. The centrifuge was then spun-down over the course of 60 s. The shorter duration of the centrifuge deceleration was related to the capabilities of the MIT centrifuge motor. Subjects reported no symptoms of motion sickness during the spin-up or spin-down process, or any other phase of the protocol, with the full motion sickness and comfort data being available elsewhere (Diaz et al., 2015b). Completion of the spin-up, transient wait, exercise period, recovery wait, and spin-down took less than 25 min.

Each exercise period lasted 15 min, and it included three exercise intensities and transitions between them. The work rates tested were: 25 W (“warm-up” intensity during 3 min), 50 W (“low” intensity during 5 min), and 100 W (“high” intensity during 5 min). The exercise work rates were selected to provide a broad range of cardiovascular responses and were based on preliminary data. Changes between workload levels were implemented linearly and smoothly, as indicated in Figure 2, to avoid potential injuries. The exercise protocol was created using the Lode Ergometer Manager, Version 9.4.4 (LEM, 2013, Groningen, Netherlands) software package provided with the ergometer. It ran automatically without any intervention from the subject or the operator. In order to avoid confounding factors, subjects were instructed to keep a constant pedal cadence of 60 rpm that was maintained using a metronome. The pedal cadence was also important for the development and validation of a cardiovascular model associated to this investigation and to be reported in separate publications (Diaz Artiles et al., 2016).

### Subjects and Study Approval

Twelve healthy subjects (6 males, 6 females) between 23 and 29 years old participated in the study. Given the important changes of the cardiovascular system with age, the age range of selected subjects was limited as much as possible to avoid age-related confounding factors. Average (±standard deviation) age and weight were 25.1 ± 2.1 years, and 69.3 ± 11.6 kg, respectively. Selected subjects exercised regularly and were comfortable engaging in continuous aerobic exercise for at least 1 h. Prior to participating in the study, subjects were asked to complete a questionnaire designed to identify exclusion criteria such as recent musculoskeletal injuries, joint or muscle pain, and cardiovascular defects or conditions. All subjects were able to complete the protocols and experienced no adverse effects. The study protocol was approved by the Committee on the Use of Humans as Experimental Subjects at MIT. Each subject received written and verbal explanations of the study protocols and gave written informed consent to participate in the experiment.

### Data Analysis and Statistics

Before analysis, raw cardiovascular signals were filtered using a low-pass Butterworth filter (cut-off frequency between 2 and 8 Hz), both in forward and reverse directions to compensate for phase delays. Additionally, data segments presenting motion artifacts were eliminated from the dataset and not included in the analysis.

Each of the cardiovascular variables was averaged over the last 2 min of each protocol phase. Thus, at each AG level, five values were calculated for each CV variable, per subject, corresponding to the baseline period (BL), and workload intensities of 0 W (centrifugation but no exercise), 25, 50, and 100 W. To study the effects of centrifugation alone (i.e., without exercise), paired, two-sided *t*-tests were used to compare the CV variables at BL with centrifugation (0 W). Since three AG conditions were tested, we implemented a Bonferroni correction for comparisons of multiple groups and used α = 0.05/3 = 0.017 to determine statistical significance. To study the effects of centrifugation combined with exercise, a two-way repeated-measures ANOVA was implemented using AG level (0, 1, and 1.4 G) and workload intensity (0, 25, 50, and 100 W) as fixed, within-subjects factors; and gender as a between-subjects factor. All the necessary data assumptions were checked and the Greenhouse–Geisser correction was applied when the data violated the assumption of sphericity (Abdi, 2010). Most of the ANOVAs resulted in significant interaction effects (CO, SV, HR, MBP, and DBP), and therefore the “simple main effects” (difference between groups at each level of each factor; for example the difference between AG levels at 100 W) are also calculated for all variables. The Bonferroni correction for multiple comparisons of the various subgroups was also incorporated in this analysis.

To further quantify and model the effects of AG and exercise work rate (WR) on the cardiovascular variables, we used the following mixed hierarchical regressions to generate dose-response curves between 0 and 1.4 G:

$${\left(\text{CV Variable}\right)}_{ij}=\rho_{i}+\beta_{1}({AG}^{2})+\beta_{2}(AG)+\beta_{3}({WR}^{2})++\beta_{4}(WR)+\beta_{5}({AG}^{*}WR)+\varepsilon_{ij}$$

where the measured cardiovascular variable (CV Variable) from the *j*^th^ measurement combination (3 AG × 4 WR levels: j = 1–12) in the *i*^th^ subject is a function of the AG term (0, 1, or 1.4 G), the workload intensity (0, 25, 50, or 100 W), and the interaction term (AG ^∗^ WR). The regression model has subject-dependent intercepts (i.e., biases; ρ*_i_*, where *i* = 1–12 subjects). In a first step, the subjects’ random effects were estimated (ρ*_i_*), accounting for the within-subject experimental design. In the second step, the CV Variable being analyzed was regressed on the rest of the terms. We tested model assumptions, including normality of the error distribution (ε*_ij_*) and homogeneity of variances. Statistical tests were performed using SYSTAT 13 Version 13.00.05 (SYSTAT Software Inc. 2009, San Jose, CA, United States).

## Results

**FIGURE 3:**
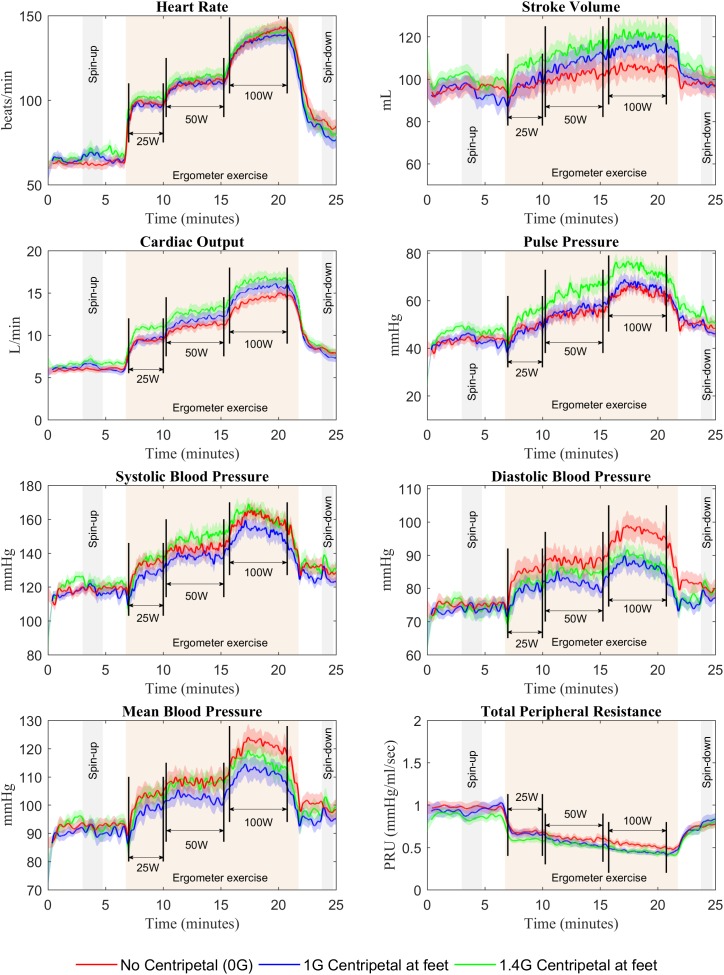
Cardiovascular responses from 12 subjects (mean ± SE) during the 25-min centrifuge run under three different AG-levels (0, 1, and 1.4 G). The protocol includes a spin-up phase (starting at Time = 3 min), the exercise phase with multiple workload intensities (from Time = 6:45 min to Time = 21:45 min), and the spin-down phase (starting at Time = 23:45 min).

Continuous recordings from the cardiovascular variables gathered during the centrifuge runs are shown in Figure 3. Each graph contains three cardiovascular responses for the 12 subjects (mean ± SE) corresponding to the three AG level experienced (0, 1, and 1.4 G). In addition, the spin-up phase (starting at Time = 3 min) and spin-down phase (starting at Time = 23:45 min), as well as the exercise phase (from Time = 6:45 min to Time = 21:45 min) are also indicated in all figures. Calculated averages (mean ± SE, including all 12 subjects) for the eight cardiovascular variables collected in each AG condition are summarized in Table 1.

**Table 1:** Calculated averages [mean (SE), including all 12 subjects] during each protocol phase for the 8 CV variables recorded during the centrifuge runs.

AG	Phase	HR (bpm)	SV (mL)	CO (L/min)	PP (mmHg)	SBP (mmHg)	DBP (mmHg)	MAP (mmHg)	TPR (PRU)
0G	BL	62.6 (2.8)	94.8 (4.4)	5.9 (0.3)	42.7 (2.1)	118.0 (3.2)	75.3 (1.8)	92.7 (2.1)	0.98 (0.06)
	0 W	63.5^b,c,d^ (3.0)	96.5 (4.3)	6.1^b,c,d^ (0.3)	44.3^c,d^ (2.1)	119.6^b,c,d^ (3.1)	75.3^b,c,d^ (1.7)	93.1^b,c,d^ (2.2)	0.96^b,c,d^ (0.06)
	25 W	98.1^a,c,d^ (2.7)	97.1^§,c^ (5.3)	9.4^§,a,c,d^ (0.4)	49.0^c,d^ (1.9)	134.3^a,c,d^ (3.8)	85.3^a,d^ (3.4)	103.6^a,c,d^ (3.4)	0.68^a,c,d^ (0.04)
	50 W	111.0^a,b,d^ (3.4)	102.8^§,b^ (5.2)	11.3^§,a,b,d^ (0.5)	55.4^a,b,d^ (2.6)	143.2^a,b,d^ (4.6)	87.9^a,d^ (3.2)	108.3^a,b,d^ (3.4)	0.59^a,b,d^ (0.04)
	100 W	141.6^‡,a,b,c^ (4.8)	102.7^§^ (5.2)	14.3^§,a,b,c^ (0.5)	62.0^a,b,c^ (1.9)	159.0^a,b,c^ (3.9)	97.0^‡,§,a,b,c^ (3.4)	121.1^‡,a,b,c^ (3.6)	0.52^a,b,c^ (0.03)
1G	BL	64.1 (2.6)	96.2 (4.0)	6.1 (0.3)	43.4 (2.0)	116.6 (1.9)	73.2 (1.2)	90.4 (1.4)	0.93 (0.07)
	0 W	64.5^§,b,c,d^ (2.7)	91.0^c,d^ (4.5)	5.8^§,b,c,d^ (0.4)	42.4^c,d^ (2.6)	116.5^b,c,d^ (3.4)	74.2^b,c,d^ (2.0)	90.5^b,c,d^ (2.4)	0.98^b,c,d^ (0.07)
	25 W	97.0^§,a,c,d^ (3.0)	98.8^c,d^ (6.0)	9.5^§,a,c,d^ (0.6)	48.2^c,d^ (3.1)	128.1^a,c,d^ (3.9)	80.0^a^ (2.5)	98.6^a,d^ (3.0)	0.66^a,c,d^ (0.05)
	50 W	110.3^§,a,b,d^ (3.6)	110.5^a,b^ (5.5)	12.1^a,b,d^ (0.7)	57.4^a,b,d^ (2.8)	138.1^a,b,d^ (4.1)	80.7^a,d^ (2.1)	101.8^a,d^ (3.1)	0.53^a,b,d^ (0.04)
	100 W	138.1^†,a,b,c^ (5.1)	114.8^a,b^ (5.0)	15.8^a,b,c^ (0.8)	65.0^a,b,c^ (2.9)	151.0^a,b,c^ (5.0)	86.0^†,a,c^ (2.8)	110.7^†,a,b,c^ (3.7)	0.44^a,b,c^ (0.03)
1.4G	BL	64.9 (2.9)	101.0 (3.4)	6.5 (0.3)	47.6 (1.7)	123.4 (2.8)	75.8 (1.9)	94.4 (2.1)	0.90 (0.05)
	0 W	69.3^∗,‡,b,c,d^ (3.4)	98.2^b,c,d^ (4.1)	6.7^‡,b,c,d^ (0.4)	47.2^b,c,d^ (2.2)	121.7^b,c,d^ (3.4)	74.5^b,c,d^ (2.5)	92.8^b,c,d^ (2.8)	0.86^b,c,d^ (0.05)
	25 W	101.6^‡,a,c,d^ (3.7)	107.9^†,a,c,d^ (4.1)	10.9^†,‡,a,c,d^ (0.4)	54.9^a,c,d^ (1.8)	136.6^a,c,d^ (3.3)	81.7^a^ (2.7)	103.2^a^ (3.2)	0.59^a,c,d^ (0.04)
	50 W	114.4^‡,a,b,d^ (4.1)	115.5^†,a,b^ (5.1)	13.2^†,a,b,d^ (0.6)	66.3^a,b^ (3.2)	151.0^a,b^ (4.8)	84.7^a^ (2.2)	108.1^a^ (3.0)	0.52^a,b,d^ (0.03)
	100 W	140.2^a,b,c^ (5.0)	120.3^†,a,b^ (5.9)	16.7^†,a,b,c^ (0.7)	71.5^a,b^ (3.0)	158.7^a,b^ (5.7)	87.2^†,a^ (3.2)	113.8^a^ (4.2)	0.44^a,b,c^ (0.04)


*Cardiovascular variables: HR, heart rate (beats per minute); SV, stroke volume (mL); CO, cardiac output (L/min); PP, pulse pressure (mmHg); SBP, systolic blood pressure (mmHg); DBP, diastolic blood pressure (mmHg); MAP, mean arterial pressure (mmHg); TPR, total peripheral resistance (Peripheral Resistance Units, PRU = mmHg/mL/s). Protocol phases: BL, baseline (no exercise or AG); 0 W, centrifugation alone; 25 W, exercise at 25 W and AG; 50 W, exercise at 50 W and AG; 100 W, exercise at 100 W and AG.*

*Values are means (SE).*

*^∗^Different from baseline (paired sample *t*-test with Bonferroni correction alpha = 0.05/3 = 0.017).*

*^†^Different from 0 G; ^‡^Different from 1 G, ^§^ Different from 1.4 G, all within the same workload levels. (Simple main effects from two-way repeated measures ANOVA with Bonferroni correction).*

*^*a*^Different from 0 W; ^*b*^Different from 25 W, ^*c*^Different from 50 W, ^*d*^Different from 100 W, all within the same AG level. (Simple main effects from two-way repeated measures ANOVA with Bonferroni correction).*

### Centrifugation Alone

A paired, two-sided *t*-test revealed a significant difference in HR between BL and centrifugation alone (0 W) at 1.4 G condition. At this AG level, HR increased by 6.8%, from 64.9 bpm at baseline to 69.3 bpm during centrifugation, and this increase was statistically significant [*t*(11) = 2.833, *P* = 0.016]. Further testing did not reveal significant differences between other averaged values at BL and centrifugation alone (0 W) for any of the AG conditions. Despite the absence of additional statistical significance, transient cardiovascular changes showed the expected tendencies in response to gravitational stress. For example, SV in 1 and 1.4 G conditions (Figure 3) decreased when starting the centrifugation phase (from 97.4 to 90.4 mL at 1 G and from 102.6 to 94.9 mL at 1.4 G), and HR increased (from 63.1 to 69.2 bpm at 1 G and from 63.5 to 73.1 bpm at 1.4 G) as well as TPR (from 0.87 PRU to 1.03 PRU at 1 G and from 0.81 PRU to 0.89 PRU at 1.4 G) as compensatory mechanisms to maintain homeostasis. As expected, no changes were observed in the 0 G condition, since subjects were not being centrifuged. We confirmed that, for each CV variable, all three baselines (0, 1, and 1.4 G) were not statistically different from each other using a one-way repeated-measures ANOVA (for all CV variables: *p* > 0.05).

### Centrifugation and Exercise

**FIGURE 4:**
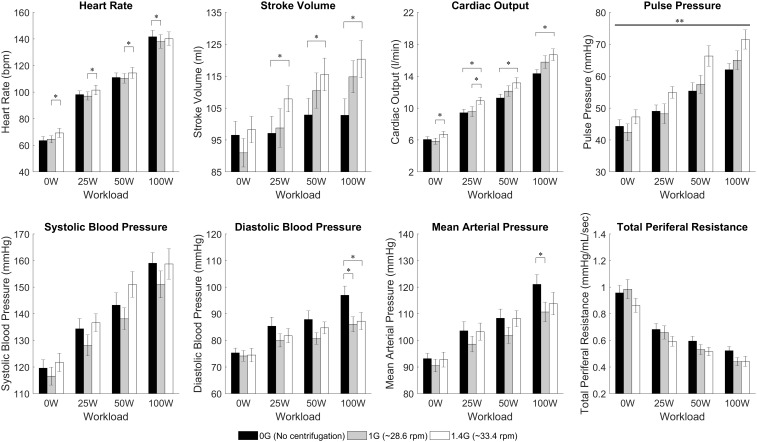
Cardiovascular data from 12 subjects (mean ± SE) by AG-level (0 G, 1 G, and 1.4 G) and workload intensity (0 W, 25 W, 50 W, and 100 W). The figure highlights significant differences between AG-levels within the same workload intensity in HR, SV, CO, DBP, and MAP (simple main effects from 2-factor repeated measures ANOVA with Bonferroni correction, ^∗^significantly different at *p* < 0.05). Our analysis also showed a statistically significant overall effect of AG-level on PP (main effects two-way repeated-measures ANOVA, ^∗∗^significantly different at *p* < 0.05) but the simple main effects did not yield a significant result. For clarity, significant differences between work rates have not been included.

Two-way repeated-measures ANOVAs were conducted to examine the effects of AG level and workload intensity on cardiovascular responses. There were statistically significant effects of AG level on PP [*F*(2,22) = 4.147, *P* = 0.03], and of workload intensity on PP [*F*(1.578,17.353) = 53.697, *P* < 0.0005], SBP [*F*(1.402,15.419) = 54.729, *P* < 0.0005], and TPR [*F*(1.465,16.111) = 160.995, *P* < 0.0005]. In these variables the interaction terms were not statistically significant. However, there was a statistically significant interaction between AG level and workload intensity on the rest of the cardiovascular variables: CO [*F*(3.037,33.408) = 4.949, *P* = 0.006], SV [*F*(6,66) = 7.867, *P* < 0.0005], HR [*F*(6,66) = 5.183, *P* < 0.0005], MBP [*F*(6,66) = 2.375, *P* = 0.039], and DBP [*F*(6,66) = 4.561, *P* = 0.001]. Therefore, for these (and also the rest) CV variables the simple main effects were calculated, and statistical results are summarized in Table 1 and Figure 4. Results from this analysis show that increasing AG level from 0 to 1.4 G significantly increases SV and CO when doing ergometer exercise at 25 W (SV: *P* = 0.044; CO: *P* = 0.016), 50 W (SV: *P* = 0.029; CO: *P* = 0.017) and 100 W (SV: *P* = 0.003; CO: *P* = 0.005). CO also increased from 1 to 1.4 G at 0 W (*P* = 0.004) and at 25 W (*P* = 0.015). Statistical analysis also revealed a significant increase in HR from 1 to 1.4 G at 0 W (*P* = 0.007), 25 W (*P* = 0.016), and 50 W (*P* = 0.018), as well as a decrease from 0 to 1 G when exercising at 100 W (*P* = 0.005). Finally, statistically significant changes in blood pressure also occurred when exercising at 100 W between 0 and 1 G (MAP: *P* = 0.029; DBP: *P* = 0.016), and between 0 and 1.4 G at 100 W (DBP: *P* = 0.039). No statistically significant simple main effects of AG-level were found on SBP, PP, or TPR. Concerning workload intensity, simple main effects were found to be statistically significant in all CV variables in almost all group combinations (see Table 1).

### Dose-Response Curves

**FIGURE 5:**
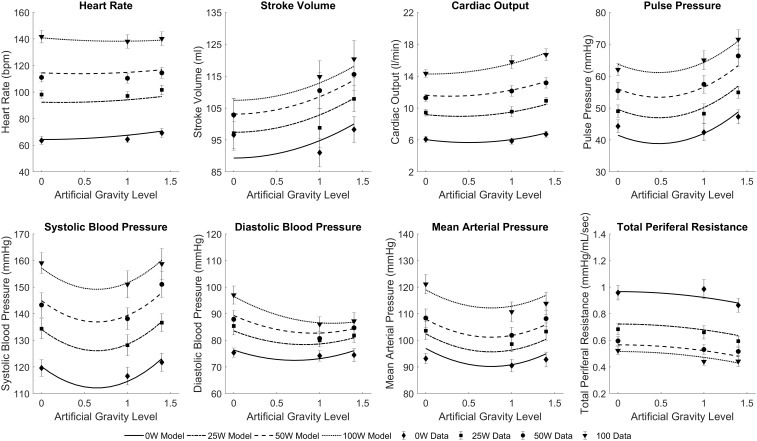
Statistically significant mixed regression models fitted to the cardiovascular experimental data across all conditions: three AG-levels (0 G, 1 G, and 1.4 G) and four workload intensities (0 W, 25 W, 50 W, and 100 W). Symbols and error bars correspond to experimental data from 12 subjects (mean ± SE) in each condition.

The results of the regression model (Equation 1) applied to the averaged cardiovascular data are given in Table 2. Only the significant β coefficients were included in the regression models (*P* < 0.05), and further interaction terms (not shown) were not significant. This specific regression analysis shows that AG level contributes to changes in all CV variables, either directly (β_1_ and β_2_) and/or through the interaction term (β_5_). Specifically, we found statistically significant quadratic relationships between AG level and all CV variables, which could be an indication of the non-linear nature of short-radius centrifugation. Results show that CO, SV, and PP generally increase with AG, especially between 1 and 1.4 G. HR increases with AG level at 0 W but this AG effect seems to disappear at higher work rates. Workload intensity has a significant effect on all variables. The positive β_4_ coefficients for PP, SV, CO, MAP, SYS, DIA, and HR indicate that these variables increase with workload intensity, and the negative β_3_ coefficient for PP, SV, CO, SYS, DIA, and HR indicates a limitation of this increase at higher work rates. Likewise, the negative β_4_ coefficient for TPR indicates that TPR decreases with increasing workload intensity; the positive β_3_ coefficient indicates that this reduction is less important at higher work rates. The interaction term was found significant and positive for CO (β_5_ > 0), and significant and negative for HR and DBP (β_5_ < 0). Experimental data and the statistical models fitted to the data are shown in Figure 5.

**Table 2:** Regression analysis coefficient for all cardiovascular variables based on the following equation:

${\left(\text{CV Variable}\right)}_{ij}=\rho_{i}+\beta_{1}({AG}^{2})+\beta_{2}(AG)+\beta_{3}({WR}^{2})++\beta_{4}(WR)+\beta_{5}({AG}^{*}WR)+\varepsilon_{i\mathrm{j.}}$						

	ρ_i_	β_1_	β_2_	β_3_	β_4_	β_5_
HR (bpm)	64.3 (bpm)	3.23 (bpm/g^2^)	NS	-4.76 10^-3^ (bpm/W^2^)	1.24 (bpm/W)	-0.058 (bpm/Wg)
SV (mL)	89.3 (mL)	5.45 (mL/g^2^)	NS	-1.89 10^-3^ (mL/W^2^)	0.37 (mL/W)	NS
CO (L/min)	6.0 (L/min)	1.47 (L/min/g^2^)	-1.47 (L/min/g)	-5.77 10^-3^ (L/min/W^2^)	0.14 (L/min/W)	0.013 (L/min/Wg)
PP (mmHg)	41.4 (mmHg)	11.62 (mmHg/g^2^)	-11.05 (mmHg/g)	-1.35 10^-3^ (mmHg/W^2^)	0.36 (mmHg/W)	NS
SBP (mmHg)	120.1 (mmHg)	19.30 (mmHg/g^2^)	-24.91 (mmHg/g)	-2.51 10^-3^ (mmHg/W^2^)	0.62 (mmHg/W)	NS
DBP (mmHg)	76.3 (mmHg)	7.66 (mmHg/g^2^)	-10.91 (mmHg/g)	-1.15 10^-3^ (mmHg/W^2^)	0.32 (mmHg/W)	-0.067 (L/min/Wg)
MAP (mmHg)	96.9 (mmHg)	11.69 (mmHg/g^2^)	-17.83 (mmHg/g)	NS	0.22 (mmHg/W)	NS
TPR (PRU)	0.97 (PRU)	-43.92 10^-3^ (PRU/g^2^)	NS	0.07 10^-3^ (PRU/W^2^)	-11.503 10^-3^ (PRU/W)	NS


*AG, artificial gravity level; WR, work rate. Only the significant parameters (*p* < 0.05) were included in the regression. A graphical representation of these results is shown in Figure 5. NS, non-significant.*

### Gender Differences

**FIGURE 6:**
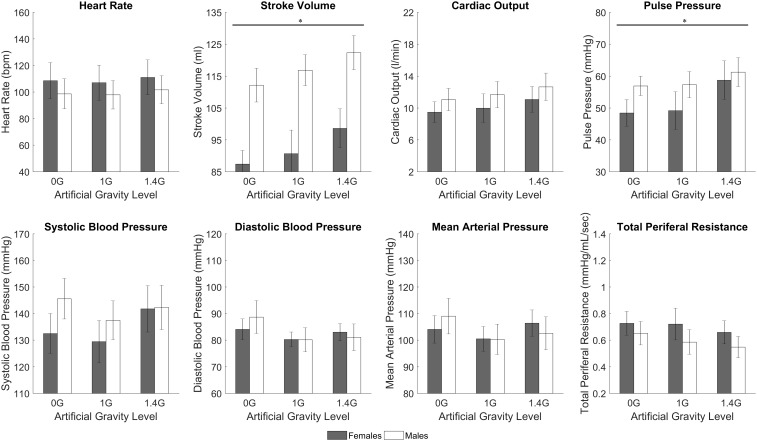
Male and female cardiovascular variables (mean ± SE) during the centrifugation runs, shown at each AG-level across all workload intensity conditions. There was a statistically significant gender effect (between-subjects effect of repeated measures ANOVA, ^∗^significantly different at *p* < 0.05) in SV (*p* < 0.0005) and PP (*p* = 0.006). Trends are also present in other variables, namely HR, CO, and TPR.

While the study was not specifically designed to study gender differences, we conducted an exploratory analysis to determine if there was significant differences between genders. We found significant differences during centrifugation between males and females (between-subjects effect of repeated measures ANOVA) in two CV variables: SV (*p* < 0.0005), and PP (*p* = 0.006). We also compared baseline measurements between both genders (two-tailed, two sample *t*-test) and found significant differences in SV (*p* < 0.0005), CO (*p* = 0.018), PP (*p* = 0.025), and SBP (*p* = 0.022). Male and females cardiovascular data at baseline and during centrifugation are shown in Table 3 and Figure 6.

**Table 3:** Cardiovascular variables [mean (SE), 6 females and 6 males] at baseline and during centrifugation separated by gender.

	Gender	Baseline	Gender effect at baseline	0 G	1 G	1.4 G	Gender effect during centrifugation
HR (bpm)	Females	65.6 (2.5)	*p* = 0.274	108.5 (13.6)	107.0 (13.2)	111.0 (13.1)	*p* = 0.179
	Males	62.1 (1.9)		98.6 (11.3)	98.0 (10.7)	101.7 (10.6)	
SV (mL)	Females	88.0 (2.7)	*p* < 0.0005	87.4 (4.2)	90.7 (7.4)	98.6 (6.1)	*p* < 0.0005
	Males	106.6 (1.9)		112.2 (5.3)	116.8 (4.9)	122.3 (5.3)	
CO (L/min)	Females	5.7 (0.3)	*p* = 0.018	9.5 (1.3)	10.0 (1.8)	11.1 (1.6)	*p* = 0.067
	Males	6.6 (0.2)		11.1 (1.4)	11.7 (1.6)	12.7 (1.7)	
PP (mmHg)	Females	42.1 (1.6)	*p* = 0.025	48.4 (4.2)	49.2 (5.9)	58.7 (6.0)	*p* = 0.006
	Males	47.1 (1.4)		56.9 (3.0)	57.3 (4.1)	61.2 (4.5)	
SBP (mmHg)	Females	115.8 (1.9)	*p* = 0.022	132.5 (7.5)	129.4 (7.9)	141.7 (8.7)	*p* = 0.078
	Males	122.9 (2.3)		145.6 (7.6)	137.4 (7.3)	142.3 (8.3)	
DBP (mmHg)	Females	73.7 (0.8)	*p* = 0.280	84.1 (3.9)	80.3 (2.7)	83.0 (3.2)	*p* = 0.810
	Males	75.8 (1.8)		88.7 (6.2)	80.1 (4.5)	81.1 (5.0)	
MAP (mmHg)	Females	91.6 (1.1)	*p* = 0.448	104.0 (5.2)	100.5 (4.7)	106.4 (4.9)	*p* = 0.932
	Males	93.3 (1.9)		109.0 (6.7)	100.3 (5.7)	102.6 (6.2)	
TPR (PRU)	Females	1.0 (0.05)	*p* = 0.061	0.73 (0.09)	0.72 (0.12)	0.66 (0.09)	*p* = 0.116
	Males	0.9 (0.04)		0.65 (0.09)	0.59 (0.09)	0.55 (0.08)	


*Female and male data were compared at baseline (two-tailed, two sample *t*-test) and during centrifugation (between-subjects effects from repeated-measures ANOVA). Results show significant differences in SV, CO, PP, and SBP at baseline and in SV and PP during centrifugation.*

## Discussion

Artificial gravity combined with exercise is a promising countermeasure to mitigate human deconditioning in space, particularly during future long duration spaceflight missions beyond Low-Earth Orbit. The use of a compact-radius centrifuge on-board might present an affordable approach to generate gravity in space. However, many questions still remain unanswered regarding the appropriate configuration, parameters, and exercise protocols to keep astronauts in a healthy physiological state. We implemented a comprehensive experimental study on human physiology during ergometer exercise using a new configuration of the MIT compact-radius centrifuge, which experienced several modifications in order to be compatible with a future use in the ISS. This experiment contributed to the identification and quantitative characterization of the short-term cardiovascular response to different AG levels and exercise workload intensities. To our knowledge, this is the first experiment that covered multiple exercise and AG levels, including a reference point with no AG.

The amplitude of the cardiovascular responses adapted to the stress level generated, not only by the exercise intensity, but also by the AG level to which the subjects were exposed while exercising. We did find a statistically significant effect of AG-level on PP (main effects two-way ANOVA), which seems to be driven by the reduction in DBP at higher gravitational levels. The average increase across exercise conditions of PP per unit of AG was 5.22 mmHg/g. Additionally, based on the simple main effect and subsequent regression analyses, CO, SV, and HR also increased with AG-level, showing an averaged increment across exercise conditions of 1.4 L/min/g, 7.6 mL/g, 2.0 bpm/g, respectively. Thus, if we consider total blood circulation (in L) an overall index of cardiovascular stress after exercise of a certain duration and intensity, the addition of centrifugation reduces the amount of exercise needed to achieve a similar degree of cardiovascular stress due to exercise alone. For example, a regular 30-min exercise with an average CO of 10 L/min generates a total blood circulation of 300 L. Our results suggest that adding centrifugation at 1 G (which increases CO to ∼11.4 L/min) reduces the exercise length an average of 12.3% to achieve the same amount of total blood circulation. Likewise, the exercise protocol is reduced an average of 16.5% when centrifugation at 1.4 G is superposed to exercise. In a similar manner, the addition of centrifugation could increase the overall index of cardiovascular stress during a specific exercise protocol of a fixed duration. Thus, superposing 1 and 1.4 G to a 30-min exercise protocol increases the total blood circulation in 14% and 19.6% respectively. While the cardiovascular index used does not capture all the complex physiological mechanisms occurring during exercise, it provides a high-level, quantitative “score” of overall cardiovascular activity, and our results suggest that centrifugation combined with exercise increases this activity with respect to exercise alone. We believe that the addition of AG during exercise protocols could be beneficial during spaceflight, providing additional stress to the cardiovascular system and/or reducing the amount of exercise currently executed during space missions.

The expected cardiovascular dynamics induced by the immediate exposure to AG are well captured during the spin-up phase. Similar to standing up in Earth’s gravity, the application of AG introduces a new stress condition that the cardiovascular system needs to overcome in order to assure the appropriate amount of blood flow to all parts of the body. We expect that the orthostatic stress introduced with centrifugation will cause multiple responses in the cardiovascular system. One of the consequences of applying a gravitational stress (applied in the head-to-toe direction) to the human body is blood pooling in the veins of the lower part of the body, which can cause an important, rapid, and transient decrease in blood pressure, particularly if the gravity stress is introduced in a suddenly manner. When there is a significant blood pressure drop, the autonomic nervous system inhibits parasympathetic activity and increases sympathetic stimulation triggered principally by the arterial baroreceptors in the carotid sinus and the aortic arch areas, which are the primary receptors for short-term control of the cardiovascular system. An increase in heart rate and contractility of the heart as well as an increase in TPR (amongst other mechanisms) will act to maintain the appropriate amount of blood flow to all the organs, in particular the brain.

In our experiment, subjects were exposed gently (over 120 s) to two different levels of AG. Our heart rate data showed an increase during the spin-up phase, and this response was more prominent, sustained, and statistically significant in the 1.4 G condition, presumably due to the cardiovascular response to a higher AG environment. Our experimental data also captured the expected dynamics of the vascular resistance: an initial decrease at the beginning of the spin-up process when first exposed to a new gravitational environment, followed by a relatively constant increase due to the sympathetic component of the cardiovascular control system. As a result of these control actions, other cardiovascular variables such as blood pressure, PP, SV, and CO showed only small changes during this phase. This is consistent with previous short-radius centrifugation studies where subjects did not show differences in pressure numerics (MAP, SBP, DBP), between baseline in supine position, 1 G, or 2 G (measured at the feet) (Goswami et al., 2015a; Verma et al., 2018). With the exception of the gravity gradient, which is particularly pronounced during short-radius centrifugation, this follows the same overall cardiovascular mechanisms and responses that someone on Earth experiences when changing from supine to upright position.

Overall, we did not find significant changes in the averaged values in any of the CV variables between BL and centrifugation alone (except HR at 1.4 G), most likely due to a combination of the specific subjects’ positioning, the muscle-pump effect, the strong gravity gradient, and the relatively low gravity levels implemented in this experiment. The subjects’ head was located at the center of rotation and the gravity level was measured at the feet. Cardiovascular sensors, in particular the baroreceptors, are located in the upper part of the body, and therefore, they were not exposed to gravitational changes as large as the feet or, in general, the lower part of the body. In the 1.4 G condition (i.e., the gravity level at the feet was 1.4 G), the AG level at the baroreceptors was 0.32 G (assuming that these are located at 25 cm from the center of rotation). Similarly, when the feet were exposed to 1 G, the baroreceptors were subjected to just 0.23 G. However, our data did show the expected trend according to the cardiovascular regulation mechanisms and suggest that higher AG-levels (i.e., higher rotation rates AND/OR larger radii) should be investigated in future experiments. Our results are consistent with a previous short-radius centrifugation study where CV responses were not different from baseline (supine) when subjects were subjected to 0.39 G at the heart (equivalent to 1 g at the feet) whereas 0.75 G at the heart (equivalent to 2 g at the feet) provided similar CV reflexes to standing, inducing a significantly higher HR and a significantly lower SV (Goswami et al., 2015a). Limitations in our centrifuge only allowed us to investigate a maximal AG-level of 1.4 G and thus, we did not find the same degree of significance in CV responses than previous studies conducted at 2 G. However, our data show the expected trends and are always consistent with existing results.

Exercise stresses the cardiovascular system and important physiological changes occur. During intense exercise, CO can increase to six or seven times the normal values (Guyton, 1990). Muscle activation increases venous return and therefore mean systemic pressure. Autonomic stimulation increases heart rate and heart contractility. As a consequence, CO also increases to meet the new metabolic demands imposed by the exercise activity. In addition, the higher muscle metabolism causes vasodilatation, and total peripheral resistance decreases to meet the metabolic demand. These cardiovascular changes due to exercise and work rate transitions were observed across all CV variables, and simple main effects analysis showed a statistically significant effect of work rate in all CV variables between most of all group combinations. Averaged changes across AG conditions were: 0.09 L/min/W (CO), 0.17 mL/W (SV), 0.22 mmHg/W (PP), 0.74 bpm/W (HR), 0.34 mmHg/W (MAP), 0.37 mmHg/W (SYS), 0.15 mmHg/W (DIA), and -0.005 PRU/W (TPR).

Exercise increases muscle metabolism and, even though the level of sympathetic stimulation is high, vascular smooth muscle in the exercise muscles dilates to satisfy the increasing local metabolic demands (functional hyperemia). According to this mechanism, in this experiment the vascular resistance decreased during the exercise protocol. In particular, significant reductions can be observed in the 1.4 G condition at the beginning of the 25 and 50 W exercise phases. After these initial reductions, the resistance seemed to level-off, presumably due to an increase in sympathetic activity, reaching an equilibrium state between two competing mechanisms: dilation due to functional hyperemia, and contraction due to sympathetic activity. Results from the regression fit showed that vascular resistance decreases with AG level, which could be explained by the more important “internal” metabolic demand of exercising muscles at higher AG levels (Bonjour et al., 2010). Bonjour and his colleagues demonstrated that the internal metabolic power during cycling, which includes all sources of metabolic energy other than the external mechanical power, is directly proportional to gravity acceleration (Bonjour et al., 2010). In our experiment, the external mechanical power was fixed (i.e., 25, 50, 100 W), but changes in the total metabolic demand could be possible due to changes in the gravitational environment. The exact nature of those changes in the presence of a gravity gradient requires further investigation, but our results are consistent with the study mentioned above. During the exercise phase at 100 W, the vascular resistances at 1 and 1.4 G seemed to overlap and level off, probably indicating that maximal muscle dilation has been reached.

A number of short-radius centrifugation studies have been reported in which subjects were conducting ergometer exercise while being exposed to AG (Iwase et al., 2002; Evans et al., 2004; Iwasaki et al., 2005; Iwase, 2005; Stenger et al., 2007; Yang et al., 2010; Wang et al., 2011). These studies mainly focused on physiological responses before and after exposure to a specific experimental intervention (typically -6 degrees head-down bed rest, or 1–3 weeks of training) with and/or without exposure to AG + ergometer exercise. Thus, data from subjects *during* centrifugation are scarce and current data do not provide a suitable basis for comparison with our own results.

Although we do not know the exact form of the dose-response relationships between the cardiovascular variables as a function of AG level or exercise intensity, we were able to fit statistically significant linear regressions to our experimental data (Table 2). To our knowledge, this is the first time that cardiovascular data have been collected during short-radius centrifugation in such a large number of conditions (3 AG-levels and 4 exercise intensities) in a rigorous and systematic way, with the same set of subjects. Thus, our dataset is unique and constitutes a good starting point to investigate gravitational dose-responses on a centrifuge. When necessary, we used the Bayesian Information Criterion (BIC) to select between multiple model options, which could include more or less parameters from Equation 1. The BIC is a criterion for model selection among a finite set of models and it not only reward a better fitting but also penalizes when adding free parameters, since this could result in overfitting the data (Schwarz, 1978). Thus, each model option has a BIC associated, and the model with a lower BIC is preferred, which implies either fewer explanatory variables, better fit, or both. As an example and based on this criterion, we included a (*WR^2^*) term (i.e., β_3_≠0) in most of the models since this term provided a better explanation of the data even with the penalization related to the addition of an extra parameter (i.e., lower BIC). We decided to use linear models due to the small sample size (3 observations per exercise intensity), and these results should be enriched with additional data to be gathered in future experiments in order to improve the accuracy and goodness of fit of the models, especially if used for prediction purposes at higher G-levels. For example, blood pressure data (particularly SBP, but also MAP and, to a certain extent, DBP) might indicate a more complex curve fitting to capture what appears to be a systematic reduction in pressure at 1 G followed by an increase at 1.4 G (Figure 5). This pattern is harder to interpret, but we can mention several factors that could contribute to explain this behavior. First, higher AG levels generate higher sympathetic activity to counteract the gravitational stress and maintain blood pressure at appropriate levels. Additionally, the muscle pump effect during ergometer exercise is theoretically more important at higher AG levels, further facilitating the return of blood to the heart in the 1.4 G condition. This hypothesis is supported by both the larger reduction in vascular resistance (indicating a more important metabolic demand of the exercising muscles) and the higher foot forces exerted on the ergometer in the 1.4 G condition (Diaz et al., 2015b) (presumably indicating a stronger muscle pump effect). Therefore, despite the stronger blood pooling in the lower body at higher AG levels, this exercise effect, combined with the higher sympathetic activity, could contribute to explaining the higher blood pressure values in the 1.4 G condition.

Our sample size is small to conduct a comprehensive study on gender effects. However, we conducted an exploratory analysis and we found gender differences in cardiovascular responses to centrifugation. Specifically, females showed smaller SV and PP than men across all levels of centrifugation and work rates. Although not significant, we also observed trends indicating that females presented larger HR and lower CO, and larger changes in HR with respect to baseline. Females also presented lower SV, CO, PP, and SBP at baseline. This is consistent with previous studies reporting gender specific patterns in cardiovascular responses to AG (Evans et al., 2018; Masatli et al., 2018). Under orthostatic stress, women typically show higher HR and lower SV (Goswami et al., 2015a; Evans et al., 2018), and they present higher incidence of orthostatic intolerance compared to men (Platts et al., 2014). Additional studies on gender effects to AG with larger number of subjects are warranted.

The Nexfin monitor measures the arterial blood pressure waveform from which it derives beat-to-beat cardiovascular parameters, such as SV, CO, and TPR, using a set of assumptions and population-based reference values, including age-related changes in the aortic pressure-area relationship (Wesseling et al., 1993, 1995; Bogert and van Lieshout, 2005; Westerhof et al., 2009; Truijen et al., 2012). A variety of studies has been conducted to evaluate the use of non-invasive devices such as Nexfin to monitor CV responses under different conditions. Cardiovascular measurements derived from the Nexfin algorithm [based on the Modelflow algorithm (Truijen et al., 2012)] showed good correlation with standard methods including traditional blood pressure measurements (e.g., Riva-Rocci/Korotkoff) (Eeftinck Schattenkerk et al., 2009; Martina et al., 2010; Martina et al., 2012; Truijen et al., 2012; Rao et al., 2018), transpulmonary thermodilution, Doppler ultrasound, and rebreathing (Wesseling et al., 1993; Harms et al., 1999; Bogert et al., 2010; Shibata and Levine, 2011; Broch et al., 2012; van der Spoel et al., 2012; Bubenek-Turconi et al., 2013). Additional studies demonstrated that SV and CO can be estimated by photoplethysmography during open heart cardiac surgery (which involves large hemodynamics changes beyond nominal conditions) (Wesseling et al., 1993), exercise (Bartels et al., 2011), and during conditions where hydrostatic pressures are altered such as fluid challenges (Critchley et al., 2014), moderate orthostatic stress due to LBNP (Harms et al., 1999; Shibata and Levine, 2011; Goswami et al., 2015b), and exposure to hyper-gravity up to +4 Gz (Manen et al., 2015). However, some authors argue that the Nexfin system is not a satisfactory substitute for transpulmonary thermodilution techniques to monitor critical care patients (Fischer et al., 2012). Other studies pointed out potential gravity-dependent limitations of pulse contours methods (PCM) to estimate CO, compared to the rebreathing technique, during microgravity (Limper et al., 2011; Arai et al., 2013) and hyper-gravity (Arai et al., 2013) using parabolic flights. However, these same studies concluded that PCM can be used to track CO dynamics during rapid changes of acceleration profiles (Limper et al., 2011). Another study compared PCM with standard rebreathing techniques in microgravity during long duration spaceflight (>119 days in space), showing limitations presumably due to changes in parameters (i.e., increase in vascular compliance in the splanchnic circulation) affecting the aortic pressure-area relationship assumed in the Modelflow algorithm. While these are important results to consider for future spaceflight applications, our subjects were representative of the normal, healthy population and did not undergo the significant “long-term” physiological changes that astronauts experience during long duration spaceflight. Additionally, it has been shown that in healthy sedentary (i.e., non-masters athletes) young individuals, the assumed “aortic ages” within the PCM were comparable with chronological ages (Shibata and Levine, 2011), supporting the reliability of Modelflow parameters under moderate orthostatic stress conditions. Despite the fact that there are currently no studies that have confirmed the validity of this technique under conditions of altered hydrostatic profiles caused by the rotating environment and presence of a gravity gradient, we took additional measures during our experiment to eliminate this potential source of error. In particular, subjects did not use the Nexfin feature that automatically corrects for hydrostatic differences in pressure when the monitored hand is not at heart level. Instead, subjects kept theirs immobile and aligned with their heart during the entire experiment. This technique has also been used in previous centrifugation studies (Goswami et al., 2015a,b; Verma et al., 2018). While the Nexfin and other similar systems such as Portapres, Finometer, and Finapres certainly have limitations, they currently are the only methods to measure continuous arterial blood pressure and estimate other CV variables continuously and non-invasively. These devices have been used extensively in the human life science space program and ground-based analog studies (Fritsch-Yelle et al., 1994; Di Rienzo et al., 2008; Verheyden et al., 2009; Hughson et al., 2012; Stenger et al., 2013; Goswami et al., 2015a,b; Lee et al., 2015; Linnarsson et al., 2015; Morita et al., 2016; Masatli et al., 2018; Verma et al., 2018), including in conditions where hydrostatic pressures are moderately altered such as Head-Up Tilt and LBNP (Linnarsson et al., 2015; Goswami et al., 2015b). Similarly, our study induced only moderate orthostatic stress, and we are using a device that facilitates comparison with other studies in the field, which is important. Our results are also quantitatively reasonable and in agreement with previous studies (Goswami et al., 2015a; Verma et al., 2018).

Other limitations of the study are related to the facilities and the resources available to conduct the experiment. Subjects’ age was not fully representative to the current astronaut population. However, age range was limited to only 6 years to avoid potential alterations in the cardiovascular system related to age differences. Additionally, the duration of the test sessions was limited to 25 min due to structural limitations of the MIT centrifuge and to avoid excessive fatigue of subjects during the exercise protocol. Thus, the relatively short duration of the exercise conditions caused some CV variables to not reach steady state, particularly at higher work rates, and extrapolations of the results outside the timeframes tested should be done with caution. Similarly, other AG levels (both between 0–1 G, and >1.4 G, measured at the subjects’ feet) and workload intensities should be tested to expand the results outside the ranges used in this experiment. Additionally, a more exhaustive experiment and analysis should be performed to fully capture proper physiological responses during spin-down. The focus of the experiment presented herein is not the post-exercise and spin-down phase. Here, the effects of both factors (recuperation after intense exercise and centrifuge deceleration) are certainly confounded in the results, since both phases are very close together due to time and protocol constraints on the centrifuge.

## Conclusion

For the first time, we characterized and quantified continuous cardiovascular response to AG generated by a short-radius centrifuge combined with lower-body ergometer exercise. Different levels of AG and workload intensity were tested and analyzed, informing future decisions and trade-offs regarding the implementation of AG in the future. The orthostatic stress introduced by AG caused the cardiovascular system to increase its overall activity to maintain homeostasis, and these responses were in general more significant at higher AG levels and higher exercise work rates. Our results suggest that AG combined with exercise may be beneficial to cardiovascular conditioning in space, by increasing the overall cardiovascular stress during exercise protocols and/or by reducing the amount of exercise to achieve a similar degree of cardiovascular stress than exercise alone. Based on this analysis, we also recommend to explore both higher AG-levels (to elicit more pronounced CV responses), as well as AG-levels between 0 and 1 G (to fill the gap between this two conditions) in future experiments to get a more comprehensive understanding of short-radius centrifugation during exercise on the CV system.

## Author Contributions

AD-A designed and conducted the human experiments, developed the statistical model, analyzed the data, and drafted the manuscript. TH provided expertise concerning the cardiovascular aspects used in this research. Also, he contributed to the modeling and the interpretation of the experimental data, and critically reviewed and approved the final manuscript. LY has contributed through the overall supervision and management of the research project and critically reviewed the experimental results and the manuscript, and approved the final manuscript.

## Conflict of Interest Statement

The authors declare that the research was conducted in the absence of any commercial or financial relationships that could be construed as a potential conflict of interest. The handling Editor declared a past co-authorship with one of the authors AD-A.
